# Prevalence of the Sphenoidal Emissary Foramen in a Chilean Osteological Sample: Anatomical and Surgical Implications

**DOI:** 10.3390/diagnostics15212800

**Published:** 2025-11-05

**Authors:** Juan José Valenzuela Fuenzalida, Catalina Alcaíno Adasme, Trinidad Soublette Tocornal, Felipe Alvial-Ahumada, Macarena Perez Gutierrez, Alejandro Bruna-Mejias, Mathias Orellana-Donoso, Pablo Nova-Baeza, Alejandra Suazo-Santibañez, Hector Gutierrez-Espinoza, Juan Sanchis-Gimeno, Maria Piagkou, George Triantafyllou, Alexandros Samolis, José E. León-Rojas

**Affiliations:** 1 Departamento de Morfología, Facultad de Medicina, Universidad Andrés Bello, Santiago 8370146, Chilealvial813@gmail.com (F.A.-A.);; 2 Departamento de Ciencias Químicas y Biológicas, Facultad de Ciencias de la Salud, Universidad Bernardo O’Higgins, Santiago 8370993, Chile; 3 Escuela de Medicina, Universidad Finis Terrae, Santiago 7501015, Chile; 4 Department of Morphological Sciences, Faculty of Medicine and Science, Universidad San Sebastián, Santiago 8420524, Chile; 5 Faculty of Health and Social Sciences, Universidad de Las Américas, Santiago 8370040, Chile; 6 Faculty of Education, Universidad Autónoma de Chile, Santiago 7500000, Chile; 7 GIAVAL Research Group, Department of Anatomy and Human Embryology, Faculty of Medicine, University of Valencia, 46001 Valencia, Spain; juan.sanchis@uv.es; 8 Department of Anatomy, School of Medicine, Faculty of Health Sciences, National and Kapodistrian University of Athens, 11527 Athens, Greece; piagkoumara@gmail.com (M.P.);; 9 “VARIANTIS” Research Laboratory, Department of Clinical Anatomy, Masovian Academy in Płock, 09402 Płock, Poland; 10 Cerebro, Emoción y Conducta (CEC) Research Group, Escuela de Medicina, Universidad de las Américas (UDLA), Quito 170521, Ecuador

**Keywords:** sphenoidal emissary foramen, foramen vesalii, foramen venosum, sphenoid bone, variation, skull base, clinical anatomy

## Abstract

**Background:** The sphenoidal emissary foramen (SEF) is an inconstant foramen of the sphenoid bone that facilitates venous communication between the pterygoid venous plexus and the cavernous sinus. Understanding its prevalence and laterality is crucial to preventing vascular injury during skull base procedures. **Methods:** A cross-sectional observational study was conducted on 133 adult Chilean dried skulls. Each specimen was examined both internally and externally to record SEF presence and laterality. Three independent observers performed the assessments under direct lighting, achieving excellent interobserver agreement (κ = 0.87, 95% CI = 0.81–0.92). Descriptive statistics, Chi-square tests, and Cramer’s V coefficients were calculated to evaluate side dominance and effect size at a significance level of *p* < 0.05. **Results:** The SEF was present in 40.17%. Bilateral foramina were observed in 26.79%, and unilateral SEF in 13.38%. Left-sided SEF (9.12%) was more common than right-sided SEF (4.26%), showing a significant difference (*p* = 0.03; Cramer’s V = 0.19, 95% CI = 0.02–0.33). This mild but significant left-sided prevalence indicates slight directional asymmetry rather than functional lateralization. **Conclusions:** The Chilean prevalence of the SEF lies within the mid-range of international data and closely aligns with Brazilian osteological reports. Although a minor left-sided predominance was observed, the effect size was weak (Cramer’s V = 0.19), reinforcing the interpretation of the SEF as a normal morphological variability rather than a true anatomical variant. Precise preoperative identification of the SEF is crucial to reduce the risk of venous injury and avoid unintentional penetration.

## 1. Introduction

The middle cranial fossa (MCF) contains several foramina located in the greater wing of the sphenoid bone, which transmit vital neurovascular structures. The foramen spinosum (FS), for example, conveys the middle meningeal artery and vein, as well as the meningeal branch of the mandibular nerve (V3). This foramen is considered an essential landmark in MCF surgery due to its proximity to critical neurovascular elements [1]. Additionally, the foramen ovale (FO), typically located medial to the FS, allows the passage of the mandibular nerve (V3), the accessory meningeal artery, and, occasionally, the lesser petrosal nerve [2]. A detailed understanding of the morphology and possible variations in these foramina is essential for accurate planning and safe execution of skull base surgical procedures [3]. However, the presence of accessory canals in the sphenoid bone represents one of the most frequent morphological variants [4,5].

A primary concern during neurosurgical and endoscopic skull base procedures is the occurrence of morphological variations that deviate from classical textbook descriptions. Such differences can increase the risk of iatrogenic injury, complicate surgical navigation, and influence clinical outcomes. The examination of human remains from anatomical dissection rooms offers a valuable opportunity to assess the prevalence of these morphological variations. Unlike radiological studies—typically performed on living clinical patients—osteological investigations provide an unbiased representation of structural anatomy and interindividual variability.

**Figure 1:**
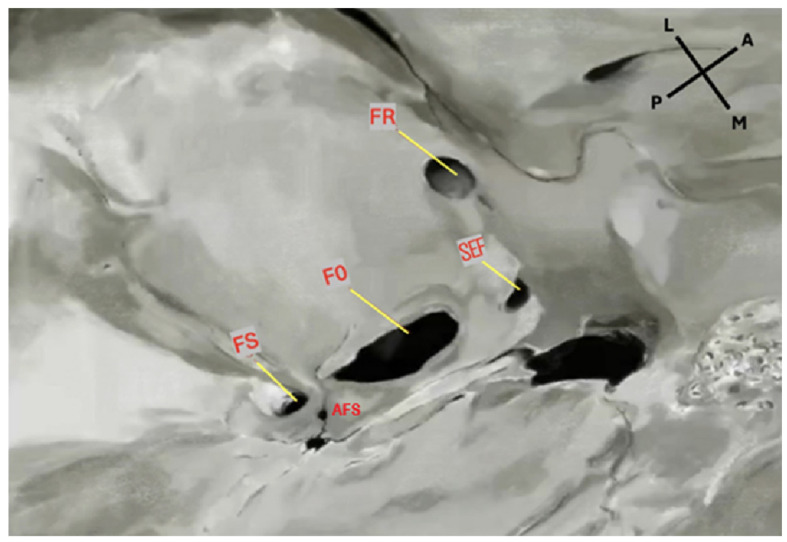
Endocranial view of the middle cranial fossa showing the foramina of the sphenoid bone. FS: foramen spinosum; FO: foramen ovale; FR: foramen rotundum; SEF: sphenoidal emissary foramen. An accessory foramen spinosum (AFS) is also observed medially to the FS. Orientation: L = lateral; M = medial; A = anterior; P = posterior. Source: Image obtained from osteological specimens after essential modification at the Anatomy Laboratories of Andrés Bello University, the University of Santiago de Chile, and Finis Terrae University, Santiago, Chile. All specimens were used exclusively for teaching and research purposes under institutional ethical approval (Resolution S:69-2024-1071).

**Figure 2:**
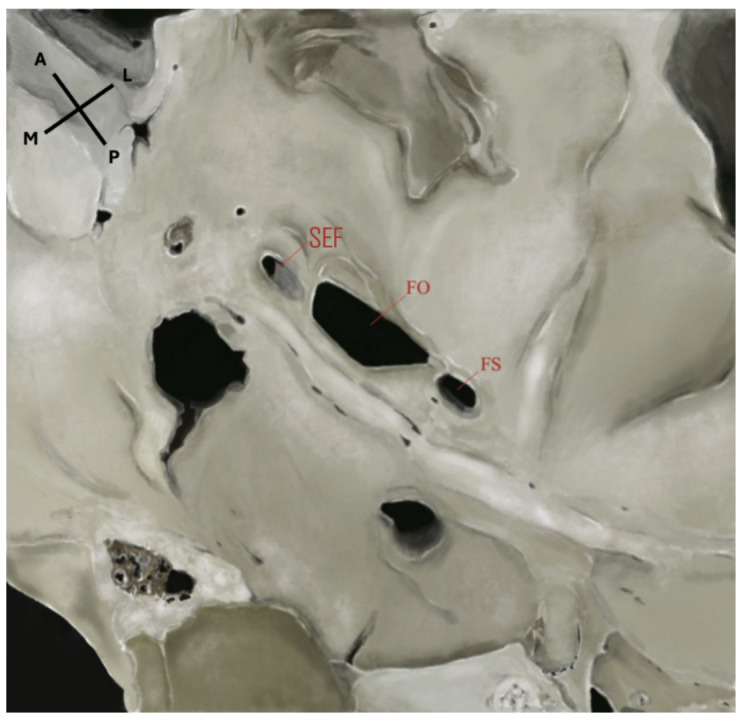
Extracranial view of the middle cranial fossa showing the foramina of the sphenoid bone. SEF: sphenoidal emissary foramen; FO: foramen ovale; FS: foramen spinosum. An accessory foramen spinosum is also visible extracranially, located medial to the FS. Orientation: A = anterior; P = posterior; L = lateral; M = medial. Source: Image obtained from osteological specimens after essential modification at the Anatomy Laboratories of Andrés Bello University, the University of Santiago de Chile, and Finis Terrae University, Santiago, Chile. All specimens were used exclusively for teaching and research purposes under institutional ethical approval (Resolution S:69-2024-1071).

The sphenoidal emissary foramen (SEF)—also known as the foramen Vesalii or foramen venosum—is an inconstant and highly variable foramen situated in the greater sphenoidal wing within the MCF (Figure 1 and Figure 2). It is usually positioned anteromedially to the FO and FS, and posteromedially to the foramen rotundum (FR). Its extracranial aperture opens into the scaphoid fossa, located within the pterygoid process [6,7]. SEF may occur unilaterally or bilaterally and, although rare, may appear duplicated. When it forms a complete canal across the cranial base and remains patent, it becomes clinically significant, as it may transmit a venous channel between the pterygoid venous plexus and the cavernous sinus (CS).

The reported prevalence of SEF varies widely across studies, ranging from 16.1% to 73.1%. Studies employing imaging modalities (such as computed tomography—CT—or cone beam CT—CBCT) generally report higher prevalence values [7,8,9,10], whereas osteological or macerated skull analyses consistently show lower frequencies [11,12,13,14,15,16]. This methodological discrepancy underscores the importance of integrating both osteological and radiological data for comprehensive anatomical interpretation. Therefore, the present study aims to determine the prevalence and laterality of the SEF in a Chilean osteological sample and to compare these findings with international reports, emphasizing their clinical and surgical relevance for skull base approaches.

## 2. Materials and Methods

### 2.1. Study Design

This study was conducted as a descriptive, cross-sectional observational investigation aimed at determining the prevalence and laterality of the SEF in a Chilean osteological sample. The research protocol adhered strictly to institutional ethical and methodological standards and was performed in accordance with the principles of the Declaration of Helsinki. The checklist was created using STROBE (Supplementary Table S1).

### 2.2. Study Population

A total of 256 human dried skulls were initially examined; 133 (47.12%) met the inclusion criteria and were retained for detailed analysis. All skulls were of unknown age and sex, as postmortem demographic information was unavailable. Because these skulls were initially prepared for educational purposes, data regarding postmortem interval or age at death could not be obtained. The specimens were sourced from the Anatomy Laboratories of Andrés Bello University, the University of Santiago de Chile, and Finis Terrae University, all located in Santiago, Chile. They formed part of institutional teaching collections used exclusively for educational and research purposes, and the study received formal approval from the institutional ethics committee (Resolution S:69-2024-1071).

The inclusion criteria required that each skull present an intact sphenoid bone with a well-preserved MCF, the absence of a calotte, allowing bilateral endocranial and exocranial examination, and clear visibility of foramina without postmortem damage or structural obstruction. Skulls showing deterioration, fragmentation, or deformation of the sphenoid bone or surrounding regions were excluded.

Additionally, ossified or completely closed foramina were excluded to prevent misclassification of non-patent structures as SEF. However, this decision may have led to a slight underestimation of true SEF prevalence, as partially ossified foramina detectable by imaging were not considered.

### 2.3. Assessments

Three trained researchers (PN, MO, and JVF) independently examined all skulls in situ on dissection tables under direct illumination using a Wood’s lamp to enhance foraminal visualization. The SEF was recorded as present when the foramen appeared completely patent to transmitted light, and as absent when the opening was incomplete, obstructed, or subdivided by a bony septum.

Each skull was systematically examined from both endocranial and exocranial perspectives, and bilateral assessments were performed to document left-sided, right-sided, or bilateral occurrence.

To ensure methodological reliability, interobserver agreement was calculated using Cohen’s Kappa coefficient (κ = 0.87, 95% CI: 0.81–0.92), indicating excellent concordance among observers.

### 2.4. Statistical Analysis

The presence, absence, and laterality (left, right, or bilateral) of the SEF were recorded for each specimen. Descriptive statistics were calculated to determine absolute frequencies, percentages, and 95% CIs for prevalence estimates. Comparative analyses of laterality (left vs. right occurrence) were performed using the Chi-square test for independence (χ^2^) to evaluate potential side dominance. A significance threshold was set at *p* < 0.05.

To quantify the magnitude of association between categorical variables, Cramer’s V coefficient was calculated as a measure of effect size. This statistic provides a standardized index of association strength, ranging from 0 (no association) to 1 (perfect association), allowing interpretation of the practical relevance of statistically significant results.

Effect size interpretation followed Cohen’s conventional thresholds, where *V* values of 0.10–0.30 indicate a weak effect, 0.30–0.50 a moderate impact, and >0.50 a strong effect. Ninety-five percent confidence intervals for Cramer’s V were also computed to provide precision estimates of the observed associations.

All statistical analyses were performed using IBM SPSS Statistics (version 29.0, IBM Corp., Armonk, NY, USA). The results were subsequently compared with previously published osteological, macerated, and radiological studies employing comparable methodologies (Table 1). Differences among study modalities (dried skulls, macerated skulls, and imaging-based analyses) were acknowledged as potential sources of methodological variability.

## 3. Results

**Figure 3:**
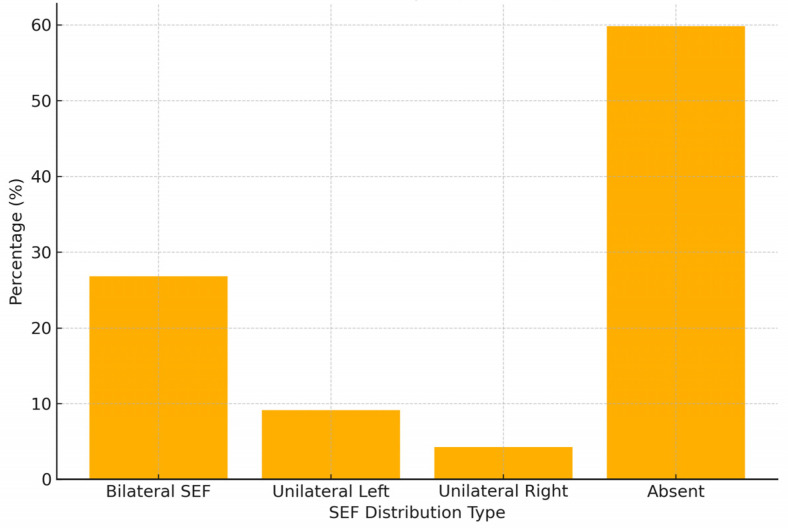
Histogram of the sphenoidal emissary foramina (SEF) distribution by laterality in the Chilean sample (n = 133). The histogram illustrates the frequency of SEF occurrence by side (left, right, bilateral, and absent). Bilateral foramina were the most common configuration (26.79%), followed by unilateral left (9.12%) and unilateral right (4.26%) cases. The majority of skulls (59.83%) did not exhibit an SEF.

A total of 133 dried skulls were analyzed, of which 40.17% (n = 54) exhibited at least one SEF. Among these, 26.79% (n = 36) displayed bilateral foramina, while 13.38% (n = 18) showed unilateral SEF occurrence. Left-sided SEF (9.12%) was more frequent than right-sided SEF (4.26%), representing a significant difference (χ^2^ = 4.68, *p* = 0.03, Cramer’s V = 0.19, 95% CI: 0.02–0.33) (Table 2, Figure 3). As illustrated in Figure 3, bilateral SEF occurred most frequently (26.79%), followed by unilateral left (9.12%) and unilateral right (4.26%) configurations. Approximately 59.83% of skulls lacked any SEF. The left-sided predominance was significant (*p* = 0.03; Cramer’s V = 0.19), indicating a weak but measurable asymmetry. This distribution pattern supports the notion of population-specific variation in SEF laterality rather than consistent directional dominance.

The sample was homogeneous in size and preservation, showing no structural asymmetry or systematic morphological bias apart from the minor left-sided predominance. When compared with international data (Table 1), substantial variability in SEF prevalence was observed, ranging from 5.0% in South African dried skulls [15] to 73.1% in Turkish CBCT studies [10]. The overall prevalence of 40.17% observed in this Chilean sample falls within the mid-range of global values and aligns closely with Brazilian osteological studies, such as the 41.6% reported by Toledo et al. [20]. Whereas osteological or macerated skull analyses consistently show lower frequencies [11,12,13,14,15,16]. This methodological discrepancy underscores the importance of integrating both osteological and radiological data for comprehensive anatomical interpretation. Therefore, the present study aims to determine the prevalence and laterality of the SEF in a Chilean osteological. 

Among comparative studies, only Leonel et al. [17] reported a significant interpopulation difference in overall SEF occurrence (*p* = 0.032), while the Chilean sample did not differ significantly from most international datasets (*p* = 0.145). A significant difference in bilateral SEF occurrence was noted only by Görürgöz et al. [10] (*p* = 0.012).

Although most previous studies did not identify right–left asymmetry, the present analysis revealed a slight but statistically significant left-sided predominance (*p* = 0.03), suggesting a population-specific trend rather than a generalized anatomical rule.

The methodological approach had a marked impact on prevalence estimates. Imaging-based studies (CT and CBCT) consistently yielded the highest mean prevalence (~54%), likely reflecting improved detection of small or partially ossified foramina. Osteological and macerated skull studies demonstrated moderate rates (~32–33%), whereas cadaveric analyses reported the lowest values (~23%), possibly due to soft tissue obscuring small emissary foramina or postmortem bone alteration.

## 4. Discussion

This observational study examined 133 dried Chilean skulls to determine the prevalence and laterality of the SEF. The results demonstrated that the SEF should be regarded as a morphological variability rather than a true variant, as it occurred with moderate frequency (40.17%) and exhibited a weak but significant left-sided predominance (*p* = 0.03; χ^2^ = 4.68; Cramer’s V = 0.19, 95% CI = 0.02–0.33). Although the effect size was small, this asymmetrical suggests a subtle directional trend rather than a functionally relevant difference. The SEF transmits the sphenoidal emissary vein (SEV), establishing a venous connection between the pterygoid plexus and the CS [21], and its variability holds clear surgical and radiological relevance.

In the Chilean sample, bilateral foramina were present in 26.79% of skulls, while unilateral foramina occurred in 13.38%. The left side was more frequently affected (9.12%) than the right (4.26%), confirming a mild population-specific asymmetry. These findings position the Chilean population within the mid-range of international reports and reinforce the concept of geographic and methodological variability in SEF occurrence.

### 4.1. Comparison with Previous Studies

When compared with international literature, SEF prevalence varied widely—from 5.0% in South African osteological samples [15] to 73.1% in Turkish CBCT studies [10]. Brazilian data (33.75–41.6%) closely mirrored the Chilean results, while Indian (36.2%), Turkish (34.8–73%), and Italian (up to 67.7%) studies reported broader ranges. Imaging-based modalities (CT and CBCT) consistently yield higher prevalence (~54%) due to their ability to visualize small or partially ossified foramina. In contrast, osteological studies report moderate frequencies (~32–33%), and cadaveric studies show lower rates (~23%) because soft tissues may obscure foramina or postmortem processes alter their margins. Only Leonel et al. [17] identified a significant interpopulation difference (*p* = 0.032), and Görürgöz et al. [10] observed significance in bilateral SEF occurrence (*p* = 0.012). In contrast to most prior studies reporting no lateral asymmetry, the present analysis detected a weak but significant left-sided predominance, likely reflecting minor developmental or vascular asymmetries rather than anatomical anomalies. Globally, unilateral SEF frequencies range from 7.2% [19] to 30.8% [10]; the 13.38% recorded here falls comfortably within this interval.

### 4.2. Clinical and Anatomical Implications

SEF carries considerable clinical importance in skull base surgery and neurovascular anatomy.

The SEV traversing this foramen can serve as a route for intracranial spread of infection—for example, CS thrombosis secondary to otitis, sinusitis, or odontogenic infection [14,17,18,19,20,21,22,23,24,25,26,27,28,29,30]. Because SEF lies in proximity to the FO, it may increase the risk of venous injury or inadvertent CS penetration during percutaneous trigeminal rhizotomy, tumor resections, or skull base drilling procedures. Moreover, SEF can act as a conduit for nasopharyngeal tumor extension into the MCF [21,23]. Radiological investigations indicate that SEF diameter tends to decline after the age of 50, potentially predisposing to venous compression and symptoms such as orbital or temporal pain [10,22].

Consequently, high-resolution imaging (CT/CBCT) remains the gold standard for identifying clinically relevant SEF, aiding in pre-operative risk assessment and navigation.

These observations highlight the necessity for individualized skull base mapping that integrates emissary and neuroforaminal anatomy to enhance surgical precision and prevent iatrogenic complications [31,32,33,34].

### 4.3. Geographic and Methodological Considerations

This study provides the first Chilean dataset on SEF prevalence, contributing novel regional insight into South American cranial variability.

Differences among Chilean, Turkish, and Indian populations may arise from genetic, morphological, or environmental factors influencing cranial venous architecture. While the use of anonymized educational skulls limited demographic stratification [35], their inclusion nonetheless broadens the global osteological record and strengthens comparative anatomical databases relevant to neurosurgery and anthropology.

### 4.4. Methodological Impact

Methodology remains a significant determinant of reported SEF frequencies.

As shown in Table 1, CT and CBCT studies often overestimate prevalence due to enhanced spatial resolution. At the same time, osteological examinations may underestimate it because of bone degradation or reduced visibility of small canals. The close agreement between the Chilean data and prior South American reports (e.g., Toledo et al. [20]) supports the methodological validity of the current findings. Nevertheless, future imaging-based validation is recommended to confirm these osteological results and explore potential sex- or age-related trends.

### 4.5. Study Strengths

This study possesses several strengths. It constitutes the first systematic assessment of the SEF in a Chilean population, thereby filling a geographical and anatomical gap in the global literature. The relatively large sample (n = 133) and strict inclusion criteria ensured robust data quality. Independent assessments by three experienced observers yielded excellent inter-observer agreement (κ = 0.87, 95% CI = 0.81–0.92), confirming high methodological reliability. The integration of anatomical, surgical, and comparative perspectives provides a comprehensive understanding of SEF variability and its clinical implications [29,35,36,37].

### 4.6. Study Limitations and Future Directions

Despite its contributions, this study has several limitations. It relied exclusively on dried skulls from educational collections, which may not represent the living population. Absence of demographic data (age, sex, ancestry) prevented correlation of SEF prevalence with biological parameters. Although osteological inspection offers precise morphological visualization, it cannot detect partially ossified or very small foramina visible through advanced imaging. The lack of soft tissues also precludes assessment of venous patency or functional significance. Finally, because no prior Chilean SEF research exists, the regional comparison remains limited. Future studies should employ multicenter CT/CBCT protocols across South American populations, linking anatomical and radiological data with clinical outcomes. Such investigations will clarify how geographic, genetic, and methodological factors shape SEF morphology and inform surgical risk assessment.

## 5. Conclusions

This study found that the SEF was present in 40.17% of Chilean skulls, with bilateral and left-sided occurrences predominating. Although the left-side difference reached statistical significance (*p* = 0.03), the effect size (Cramer’s V = 0.19) indicates that the asymmetry is weak and likely of limited clinical consequence. Recognizing this foramen is essential for avoiding vascular injury and unintentional CS penetration during skull base procedures. By contributing new regional data, this study enhances the global anatomical record. It underscores the need for future imaging-based and multicenter research to refine knowledge of SEF prevalence, patency, and surgical relevance. Accurate identification of this structure will improve diagnostic precision, operative safety, and our broader understanding of skull base venous anatomy.

## Figures and Tables

**Table 1:** Reported prevalence of the sphenoidal emissary foramen (SEF) in different populations and study modalities, DS-dried skulls, MS-macerated skulls, CBCT-Cone beam computed tomography scan, T-total, L-left, and R-right side.

Author	Year	Country	Sample/Study Type	Frequency %				
				Total (%)	Bilateral	Unilateral		
						T	L	R
Shinohara et al. [16]	2010	Brazil	400 MS	33.75	15.5	18.25	10.55	7.75
Aviles et al. [14]	2011	Mexico	25 DS	20.0	4.0	16.0	8.0	8.0
Chaisuksunt et al. [11]	2012	Thailand	377 DS	16.1	4.2	11.9	8.2	3.7
Ozer et al. [13]	2014	Turkey	172 DS	34.8	9.3	25.5	15.1	10.4
Lazarus et al. [15]	2015	South Africa	200 DS	5.0	-	-	-	-
Alves et al. [12]	2017	Brazil	178 MS	32.0	23.06	8.46	4.21	4.21
Bayrak et al. [9]	2018	Turkey	317 CBCTs	28.1	6.9	21.1	9.8	11.3
Görürgöz et al. [10]	2020	Turkey	260 CBCTs	73.1	42.3	30.8	16.2	14.65
Leonel et al. [17]	2020	Brazil	1000 CTs	46.8	25.4	21.4	9.6	11.8
			170 DS	45.2	18.8	26.4	14.7	11.7
			50 cadavers	14.0	6.0	8.0	4.0	4.0
Palamenghi et al. [7]	2023	Italy	300 CTs	67.7	-	-	-	-
Unver et al. [18]	2014	Turkey	44 DS & 18 cadavers	32.3 (31.8 & 33.3)	-	-	-	-
Raval et al. [19]	2015	Western India	150 DS	60.0	32.23	35.56	20.0	12.0
Toledo et al. [20]	2016	Brazil	84 DS	41.6	16.6	-	-	-
Present Study	2025	Chile	133 DS	40.17	26.79	13.38	9.12	4.26

**Table 2:** Prevalence and laterality of the sphenoidal emissary foramen (SEF) in the Chilean osteological sample (n = 133, total number of the sample).

SEF Occurrence Type	Frequency (n)	Percentage (%)	*p*-Value	Effect Size
				(Cramer’s V, 95% CI)
Bilateral SEF	36	26.79	0.012	0.21 (0.05–0.36)
Unilateral SEF (Left)	12	9.12	0.03	0.19 (0.02–0.33)
Unilateral SEF (Right)	6	4.26	-	-
SEF Absent	79	59.83	-	-
Total	133	100	-	-

## Data Availability

All data are available upon reasonable request to the corresponding author.

## Supplementary Materials

The following supporting information can be downloaded at: https://www.mdpi.com/article/10.3390/diagnostics15212800/s1, Table S1: Strobe Checklist.
