# Supplementary material for: Prevalence of the Sphenoidal Emissary Foramen in a Chilean Osteological Sample: Anatomical and Surgical Implications
Source: Diagnostics (Basel). 2025 Nov 5;15(21):2800. doi: 10.3390/diagnostics15212800 (PMC12609266; doi:10.3390/diagnostics15212800)
Supplement: Supplementary file 1 [file diagnostics-15-02800-s001.zip › diagnostics-3902448-supplementary.pdf]

Table suntary 1 Strobe.

STROBE Statement—Checklist of items that should be included in reports of cohort studies

	Item No	Recommendation
Title and abstract	1	Prevalence of the Sphenoidal Emissary Foramen in a Chilean Osteological Sample: Anatomical and Surgical Implications
		(b) Provide in the abstract an informative and balanced summary of what was done and what was found
Introduction		
Background/rationale	2	Explain the scientific background and rationale for the investigation being reported
Objectives	3	State specific objectives, including any prespecified hypotheses
Methods		
Study design	4	This study was conducted as a descriptive, cross-sectional observational investigation aimed at determining the prevalence and laterality of the SEF in a Chilean osteological sample. The research protocol adhered strictly to institutional ethical and methodological standards and was performed in accordance with the principles of the Declaration of Helsinki.
Setting	5	The specimens were sourced from the Anatomy Laboratories of Andrés Bello University, the University of Santiago de Chile, and Finis Terrae University, all located in Santiago, Chile.
Participants	6	A total of 256 human dried skulls were initially examined, of which 133 specimens (47.12%) met the inclusion criteria and were retained for detailed analysis. All skulls were of unknown age and sex, as postmortem demographic information was unavailable. Because these skulls were originally prepared for educational purposes, data regarding postmortem interval or age at death could not be obtained.
Variables	7	skull present an intact sphenoid bone with a well-preserved MCF, the absence of a calotte, allowing bilateral endocranial and exocranial examination, and clear visibility of foramina without postmortem damage or structural obstruction. Skulls showing deterioration, fragmentation, or deformation of the sphenoid bone or surrounding regions were excluded
Data sources/measurement	8*	This study provides the first Chilean dataset on SEF prevalence, contributing novel regional insight into South American cranial variability.<br>Differences among Chilean, Turkish, and Indian populations may arise from genetic, morphological, or environmental factors influencing cranial venous architecture.<br>While the use of anonymized educational skulls limited demographic stratification, the inclusion of these data nonetheless broadens the global osteological record and strengthens comparative anatomical databases relevant to neurosurgery and anthropology
Bias	9	Describe any efforts to address potential sources of bias
Study size	10	A total of 133 dried skulls were analyzed,
Quantitative variables	11	To quantify the magnitude of association between categorical variables, Cramer's V coefficient was calculated as a measure of effect size. This statistic provides a standardized index of association strength, ranging

		from 0 (no association) to 1 (perfect association), allowing interpretation of the practical relevance of statistically significant results. Effect size interpretation followed Cohen's conventional thresholds, where V values of 0.10–0.30 indicate a weak effect, 0.30–0.50 a moderate effect, and >0.50 a strong effect. Ninety-five percent confidence intervals for Cramer's V were also computed to provide precision estimates of the observed associations.
Statistical methods	12	The presence, absence, and laterality (left, right, or bilateral) of the SEF were recorded for each specimen. Descriptive statistics were calculated to determine absolute frequencies, percentages, and 95% CIs for prevalence estimates. Comparative analyses of laterality (left vs. right occurrence) were performed using the Chi-square test for independence (χ^2) to evaluate potential side dominance. A significance threshold was set at $p < 0.05$.
		(b) Describe any methods used to examine subgroups and interactions
		(c) Explain how missing data were addressed
Results		The sample was homogeneous in size and preservation, showing no structural asymmetry or systematic morphological bias apart from the minor left-sided predominance. When compared with international data (Table 1), substantial variability in SEF prevalence was observed, ranging from 5.0% in South African dried skulls [15] to 73.1% in Turkish CBCT studies [10]. The overall prevalence of 40.17% observed in this Chilean sample falls within the mid-range of global values and aligns closely with Brazilian osteological studies, such as the 41.6% reported by Toledo et al. [17].
Participants	13*	(a) Report numbers of individuals at each stage of study—eg numbers potentially eligible, examined for eligibility, confirmed eligible, included in the study, completing follow-up, and analysed
		(b) Give reasons for non-participation at each stage
		(c) Consider use of a flow diagram
Descriptive data	14*	(a) Give characteristics of study participants (eg demographic, clinical, social) and information on exposures and potential confounders
		(b) Indicate number of participants with missing data for each variable of interest
		(c) Summarise follow-up time (eg, average and total amount)
Outcome data	15*	Report numbers of outcome events or summary measures over time
Main results	16	
Other analyses	17	Report other analyses done—eg analyses of subgroups and interactions, and sensitivity analyses
Discussion	17	Although the effect size was small, this asymmetrical suggests a subtle directional trend rather than a functionally relevant difference. The SEF transmits the sphenoidal emissary vein (SEV), establishing a venous connection between the pterygoid plexus and the CS [18], and its variability holds clear surgical and radiological relevance.
Key results	18	Summarise key results with reference to study objectives
Limitations	19	Despite its contributions, this study has several limitations. It relied exclusively on dried skulls from educational collections, which may not represent the living population. Absence of demographic data (age, sex, ancestry) prevented correlation of SEF prevalence with biological parameters. Although osteological inspection offers precise morphological visualization, it cannot detect partially ossified or very small foramina visible through advanced imaging
Interpretation	20	
Generalisability	21	Discuss the generalisability (external validity) of the study results
		Other information. 22 None

Funding	22	Give the source of funding and the role of the funders for the present study and, if applicable, for the original study on which the present article is based
